# Supplementary material for: Identification of a Novel Therapeutic Target against XDR Salmonella Typhi H58 Using Genomics Driven Approach Followed Up by Natural Products Virtual Screening
Source: Microorganisms. 2021 Dec 3;9(12):2512. doi: 10.3390/microorganisms9122512 (PMC8708826; doi:10.3390/microorganisms9122512)
Supplement: Supplementary file 1 [file microorganisms-09-02512-s001.zip › Supplementary Table S1.pdf]

Supplementary Table S1: Proteins involved in Unique metabolic pathways

S. No	Unique Metabolic Pathways	Pathways ID	No. of Proteins	Proteins Names
**1**	Bacterial secretion system	sty03070	36	outer membrane protein TolC<br>pathogenicity island protein<br>pathogenicity 1 island effector protein<br>outer membrane secretory protein<br>pathogenicity island lipoprotein<br>type III secretion protein ssaS<br>type III secretion protein yscR<br>type III secretion protein ssaT<br>type III secretion protein invA<br>type III secretion ATP synthase<br>type III secretion protein ssaN<br>type III secretion protein ssaQ<br>type III secretion protein ssaV<br>type III secretion protein<br>surface presentation of antigens protein<br>protein-export membrane protein SecD<br>protein-export membrane protein SecF<br>preprotein translocase subunit SecE<br>protein-export membrane protein<br>preprotein translocase subunit<br>hypothetical protein<br>membrane protein insertase<br>preprotein translocase subunit SecA<br>cell division protein FtsY<br>protein-export protein SecB<br>signal recognition particle protein<br>sec-independent protein translocase protein tatA<br>sec-independent protein translocase protein tatB<br>sec-independent protein translocase protein tatC<br>sec-independent protein translocase protein tatE<br>Rhs-family protein<br>hypothetical protein<br>type VI secretion protein HCP<br>lipoprotein<br>type VI secretion protein impK

				ClpB-like protein
**2**	Two-component system	sty02020	167	phosphate regulon sensor protein PhoR<br>phosphate regulon transcriptional regulatory protein PhoB<br>phosphate ABC transporter substrate-binding protein<br>sensor protein PhoQ<br>transcriptional regulator PhoP<br>outer membrane invasion protein pagC<br>hypothetical protein pagO<br>outer membrane virulence protein pagD<br>outer membrane protease E pgtE<br>nonspecific acid phosphatase phoN<br>two-component sensor kinase EnvZ<br>two-component response regulator OmpR<br>outer membrane protein C<br>outer membrane protein F<br>two component sensor kinase rstB<br>two-component response regulator rstA<br>two-component sensor kinase cpxA<br>two-component response regulatory protein cpxR<br>efflux system protein<br>protease DO<br>two-component sensor kinase creC<br>two-component response regulator creB<br>two-component system sensor kinase baeS<br>two-component system response regulator baeR<br>efflux pump acrD<br>transporter protein mdtD<br>two-component sensor kinase basS<br>RND-family transporter protein dtB<br>efflux pump mdtA<br>two-component response regulator basR<br>two-component system sensor histidine kinase qseC<br>two-component system response regulator qseB<br>flagellar transcriptional activator FlhD<br>flagellar transcriptional activator flhC<br>flagellin fliC<br>motility protein A<br>lipopolysaccharide biosynthesis protein arnB<br>RNA polymerase sigma transcription factor for flagellar operon

				sensor protein KdpD<br>KDP operon transcriptional regulatory protein<br>potassium-transporting ATPase subunit A<br>potassium-transporting ATPase subunit B<br>potassium-transporting ATPase subunit C<br>two-component sensor protein histidine protein kinase<br>trimethylamine-N-oxide reductase<br>chaperone protein TorD<br>aerobic respiration control sensor protein<br>global response regulator<br>two-component system sensor kinase tctE<br>hypothetical proteiN tctA<br>hypothetical proteiN tctB<br>hypothetical proteiN tctC<br>chromosomal replication initiator proteiN dnaA<br>D-alanyl-D-alanine dipeptidase vanX<br>citrate lyase acyl carrier protein citD<br>citrate lyase subunit beta citE<br>citrate lyase subunit alpha<br>apo-citrate lyase phosphoribosyl-dephospho-CoA transferase<br>phosphoribosyl-dephospho-CoA transferase citX<br>2-(5''-triphosphoribosyl)-3'-dephosphocoenzyme-A synthase<br>triphosphoribosyl-dephospho-CoA transferase CitG<br>citrate carrier citT<br>two-component sensor kinase dcuS<br>two-component response regulator dcuR<br>anaerobic C4-dicarboxylate transporter dcuB<br>NAD-linked malic enzyme; malate oxidoreductase<br>nitrate/nitrite sensor protein NarX<br>nitrate/nitrite sensor protein NarQ<br>respiratory nitrate reductase 1 subunit gamma<br>respiratory nitrate reductase 2 subunit gamma<br>respiratory nitrate reductase 1 subunit delta<br>respiratory nitrate reductase 2 subunit delta<br>fumarate reductase, flavoprotein subunit frdA<br>fumarate reductase, iron-sulfur protein frdB<br>fumarate reductase complex subunit C<br>fumarate reductase complex subunit D

				regulatory protein uhpC;<br>two-component system sensor histidine kinase uhpB<br>Two-component system response regulator uhpA<br>hexosephosphate transport protein uhpT<br>RcsF protein<br>sensor protein RcsC<br>two-component system sensor kinase rcsD<br>colanic acid capsullar biosynthesis activation protein A<br>capsule synthesis protein B regulatory protein<br>sensor protein barA<br>invasion response-regulator uvrY<br>cell-division regulatory protein sdiA<br>carbon storage regulator csrA<br>transcriptional regulator fimZ<br>[protein-PII] uridylyltransferase glnD<br>nitrogen regulatory protein P-II glnB<br>two-component system sensory histidine kinase glnL<br>two-component system response regulator glnG<br>glutamine synthetase glnA<br>two-component system sensor protein zraS<br>transcriptional regulator<br>TonB-dependent outer membrane siderophore receptor protein<br>ferrienterobactin receptor<br>sensor kinase DpiB<br>sensor kinase Cita<br>transcriptional regulator CriR<br>transcriptional regulator Citb<br>[citrate (pro-3S)-lyase] ligase<br>nitrate/nitrite response regulator protein NarL<br>nitrate/nitrite response regulator protein NarP<br>respiratory nitrate reductase 1 subunit alpha<br>respiratory nitrate reductase 2 subunit alpha<br>respiratory nitrate reductase 1 subunit beta<br>respiratory nitrate reductase 2 subunit beta<br>fumarate reductase complex subunit D<br>sensor protein RcsC<br>invasion response-regulator<br>cell-division regulatory protein<br>transcriptional regulator

				wo-component system sensory histidine kinase<br>transcriptional regulator zraR<br>resistance protein zraP<br>acetyl-CoA acetyltransferase ACAT;<br>phosphoglycerate transport regulatory protein PgtC<br>phosphoglycerate transport system sensor protein PgtB<br>phosphoglycerate transporter protein pgtP<br>C4-dicarboxylate transport protein dctA<br>RNA polymerase sigma-54 factor rpoN<br>sensor kinase protein glrK<br>transcriptional regulator glrR<br>uptake hydrogenase small subunit hyaA<br>hydrogenase-2 small subunit hyaA<br>hydrogenase-2 large subunit hyaB<br>hydrogenase-1 large subunit hyaB<br>Ni/Fe-hydrogenase 1 b-type cytochrome subunit<br>methyl-accepting chemotaxis citrate transducer<br>chemotaxis protein methyltransferase<br>methyl-accepting chemotaxis protein II<br>aerotaxis receptor protein<br>chemotaxis protein CheA<br>chemotaxis protein CheY<br>receptor/regulator protein cheV<br>two-component response regulator ttrR<br>two-component response regulator ttrA<br>two-component response regulator ttrB<br>two-component response regulator ttrC<br>two-component sensor kinase ssrA<br>two-component sensor kinase ssrB<br>glutamate/aspartate transport ATP-binding protein GltL<br>glutamate/aspartate transport ATP-binding protein GltK<br>glutamate/aspartate transport ATP-binding protein GltJ<br>ABC transporter substrate-binding protein gltI<br>protein-glutamate methylesterase<br>cyclic AMP receptor protein,catabolite gene activator<br>terminal oxidase subunit I

				cytochrome d ubiquinol oxidase subunit I<br>cytochrome bd-II oxidase subunit I<br>terminal oxidase subunit II<br>cytochrome d ubiquinol oxidase subunit II<br>cytochrome oxidase subunit II<br>hypothetical protein cydX<br>outer membrane protein TolC<br>polysaccharide export protein wza<br>protein-tyrosine phosphatase wzb<br>tyrosine-protein kinase etk-wzc<br>UDP-N-acetyl-D-glucosamine 2-epimerase wecB<br>UDP-ManNAc dehydrogenase wecC<br>Pectinesterase E3.1.1.11<br>flagellin synthesis negative regulator flgM<br>tetrathionate reductase subunit A<br>tetrathionate reductase subunit B<br>outer membrane protein TolC
**3**	Quorum sensing	sty0202	57	autoinducer-2 production protein LuxS<br>RNA-binding protein Hfq<br>anthranilate synthase component I<br>3-deoxy-7-phosphoheptulonate synthase<br>3-deoxy-D-arabinoheptulosonate 7-phosphate synthase<br>phospho-2-dehydro-3-deoxyheptonate aldolase<br>two-component system sensor histidine kinase<br>two-component system response regulator<br>sensor kinase protein<br>transcriptional regulator<br>flagellar transcriptional activator FlhD<br>flagellar transcriptional activator<br>KDP operon transcriptional regulatory protein<br>cell-division regulatory protein<br>amino acid transporter<br>ABC transporter permease lsrC<br>ABC transporter permease lsrD<br>ABC transporter substrate-binding protein lsrA<br>sugar kinase lsrK<br>regulatory protein lsrR<br>aldolase lsrF<br>autoinducer-2 (AI-2) modifying protein LsrG<br>high-affinity branched-chain amino acid ABC transporter permease livM

				high-affinity branched-chain amino acid ABC transporter permease livH<br>high-affinity branched-chain amino acid ABC transporter substrate-binding protein<br>RNase II stability modulator<br>oligopeptide ABC transporter substrate-binding protein OppA<br>colanic acid capsullar biosynthesis activation protein A<br>RNase II stability modulator<br>large repetitive protein<br>long-chain-fatty-acid--CoA ligase<br>cyclic AMP receptor protein,catabolite gene activator<br>zinc uptake regulation protein<br>GTP cyclohydrolase II<br>riboflavin biosynthesis protein RibD<br>hypothetical protein<br>hypothetical protein<br>oligopeptide ABC transporter permease OppB<br>oligopeptide ABC transporter ATP-binding protein OppD<br>oligopeptide ABC transporter ATP-binding protein OppF<br>inner membrane transport protein<br>ABC transporter permease<br>ABC transporter ATP-binding protein ddpD<br>ABC transporter ATP-binding protein ddpF<br>membrane protein insertase yidC<br>preprotein translocase subunit SecE<br>protein-export membrane protein secG<br>preprotein translocase subunit secY<br>hypothetical protein yajC<br>preprotein translocase subunit SecA<br>cell division protein FtsY<br>protein-export protein SecB<br>signal recognition particle protein SRP54<br>periplasmic murein peptide-binding protein MppA<br>substrate-binding transport protein
**4**	Bacterial chemotaxis	sty02030	19	methyl-accepting chemotaxis citrate transducer<br>methyl-accepting chemotaxis protein II<br>aerotaxis receptor protein<br>maltose/maltodextrin-binding protein MalE

				D-ribose-binding periplasmic protein<br>D-galactose-binding periplasmic protein<br>periplasmic dipeptide transport protein<br>chemotaxis protein CheA<br>purine binding chemotaxis protein<br>chemotaxis protein CheY<br>chemotaxis protein CheZ<br>protein-glutamate methylesterase<br>receptor/regulator protein<br>chemotaxis protein methyltransferase<br>flagellar motor switch protein FliG<br>flagellar motor switch protein FliM<br>flagellar motor switch protein FliN<br>motility protein A<br>motility protein B
**5**	Flagellar assembly	sty02040	45	RNA polymerase sigma-70 factor<br>flagellar hook-basal body complex protein FliE<br>flagellar basal-body M-ring protein<br>flagellar motor switch protein FliG<br>flagellar assembly protein FliH<br>flagellum-specific ATP synthase<br>flagellar protein FliJ<br>flagellar hook-length control protein<br>flagellar basal body-associated protein FliL<br>flagellar motor switch protein FliM<br>flagellar motor switch protein FliN<br>flagellar protein FliO<br>flagellar biosynthetic protein FliP<br>flagellar biosynthetic protein FliQ<br>flagellar biosynthetic protein FliR<br>flagellar protein FlhE<br>flagellar biosynthesis protein FlhA<br>flagellar biosynthetic protein FlhB<br>flagellar basal body P-ring protein FlgA<br>flagellar basal-body rod protein FlgB<br>flagellar basal-body rod protein FlgC<br>flagellar hook formation protein FlgD<br>flagellar hook protein FlgE<br>flagellar basal-body rod protein FlgF<br>flagellar basal-body rod protein FlgG<br>flagellar L-ring protein<br>flagellar P-ring protein<br>flagellar protein FlgJ<br>flagellar hook-associated protein 1<br>flagellar hook-associated protein 3

				flagellin K02406 fliC; flagellin<br>flagellar hook associated protein 2<br>flagellar protein FliS<br>flagellar protein FliT<br>motility protein B<br>motility protein A<br>flagellin synthesis negative regulator<br>flagella synthesis protein FlgN<br>cystine-binding periplasmic protein<br>flagella biosynthesis regulator FliZ<br>RNA polymerase sigma-54 factor<br>flagellar transcriptional activator FlhD<br>flagellar transcriptional activator<br>RNA polymerase sigma transcription factor for flagellar operon
**6**	beta-Lactam resistance	sty01501	18	outer membrane protein F<br>outer membrane protein C<br>muropeptide transporter AmpG<br>glycosyl hydrolase<br>oligopeptide ABC transporter substrate-binding protein OppA<br>periplasmic murein peptide-binding protein MppA<br>oligopeptide ABC transporter permease OppB<br>oligopeptide ABC transporter ATP-binding protein OppD<br>oligopeptide ABC transporter ATP-binding protein OppF<br>tRNA-splicing ligase RtcB<br>outer membrane protein TolC<br>beta-lactamase<br>acriflavin resistance protein A<br>acriflavin resistance protein B<br>penicillin-binding protein 1A<br>penicillin-binding protein 2<br>penicillin-binding protein 3<br>penicillin-binding protein
**7**	Vancomycin resistance	sty01502	8	D-alanyl-D-alanine dipeptidase<br>D-alanine:D-alanine ligase B<br>D-alanine:D-alanine ligase A<br>alanine racemase ddlA<br>alanine racemase alr<br>alanine racemase dadX<br>UDP-N-acetylmuramoylalanyl-D-glutamyl-2,6-diaminopimelate--D-alan alanyl ligase

				UDP-N-acetylglucosamine:N-acetylmuramyl-(pentapeptide) pyrophosphoryl-undecaprenol N-acetylglucosamine transferase
**8**	Cationic antimicrobial peptide (CAMP) resistance	sty01503	15	sensor protein PhoQ<br>transcriptional regulator PhoP<br>KDO phosphoethanolamine transferase<br>antimicrobial peptide resistance and lipid A acylation protein<br>lipopolysaccharide biosynthesis protein arnB<br>lipopolysaccharide modification protein pmrF<br>lipopolysaccharide modification protein arnA<br>two-component sensor kinase<br>peptide ABC transporter substrate-binding protein<br>two-component response regulatory protein<br>thiol:disulfide interchange protein<br>4-deoxy-4-formamido-L-arabinose-phosphoundecaprenol deformylase<br>4-amino-4-deoxy-L-arabinose lipid A transferase<br>acyl-[acyl-carrier-protein]--UDP-N-acetylglucosamine O-acyltransferase
**9**	Phenylalanine, tyrosine and tryptophan biosynthesis	sty00400	17	3-deoxy-7-phosphoheptulonate synthase<br>3-deoxy-D-arabinoheptulosonate 7-phosphate synthase<br>phospho-2-dehydro-3-deoxyheptonate aldolase<br>3-dehydroquinate synthase<br>3-dehydroquinase<br>shikimate dehydrogenase<br>shikimate 5-dehydrogenase<br>3-phosphoshikimate 1-carboxyvinyltransferase<br>chorismate synthase<br>anthranilate synthase component I<br>tryptophan synthase subunit alpha<br>tryptophan synthase subunit beta<br>bifunctional chorismate mutase/prephenate dehydrogenase<br>decarboxylase<br>catalase<br>4-hydroxyphenylacetate 3-monooxygenase coupling protein
**10**	Taurine and hypotaurine metabolism	sty00430	3	gamma-glutamyltranspeptidase<br>phosphate acetyltransferase<br>acetate kinase

**11**	Lipopolysaccharide biosynthesis	sty00540	46	acyl-[acyl-carrier-protein]--UDP-N-acetylglucosamine O-acyltransferase<br>UDP-3-O-[3-hydroxymyristoyl] N-acetylglucosamine deacetylase 1pxC<br>UDP-3-O-[3-hydroxymyristoyl] glucosamine N-acyltransferase 1pxC<br>lipid-A-disaccharide synthase lpxB<br>tetraacyldisaccharide 4'-kinase lpxK<br>D-arabinose 5-phosphate isomerase kdsD<br>2-dehydro-3-deoxyphosphooctonate aldolase kdsA<br>3-deoxy-D-manno-octulosonate 8-phosphate phosphatase KdsC<br>3-deoxy-manno-octulosonate cytidylyltransferase kdsB<br>3-deoxy-D-manno-octulosonic acid transferase kdtA<br>lipid A biosynthesis lauroyl acyltransferase htrB<br>lipid A biosynthesis lauroyl acyltransferase ipxM<br>membrane-bound beta-hydroxylase lpxO<br>outer membrane protein lpxR<br>hypothetical protein lpxT<br>lipid A phosphoethanolamine transferase eptA<br>KDO phosphoethanolamine transferase eptB<br>antimicrobial peptide resistance and lipid A pagP<br>4-amino-4-deoxy-L-arabinose lipid A transferase<br>acyltransferase lpxP<br>phosphoheptose isomerase gmhA<br>ADP-heptose synthase gmhC<br>D,D-heptose 1,7-bisphosphate phosphatase gmhB<br>ADP-L-Glycero-D-mannoheptose-6-epimerase gmhD<br>ADP-heptose--LPS heptosyltransferase waaC;<br>ADP-heptose--LPS heptosyltransferase waaF<br>lipopolysaccharide core biosynthesis protein<br>LPS core phosphoethanolamine transferase eptC<br>lipopolysaccharide core biosynthesis protein glycosyltransferase waaH

				lipopolysaccharide core biosynthesis protein way<br>3-deoxy-D-manno-oct-2-ulosonate III transferase WaaZ<br>lipopolysaccharide core biosynthesis protein<br>lipopolysaccharide 1,6-galactosyltransferase<br>lipopolysaccharide 1,2-N-acetylglucosaminetransferase<br>lipopolysaccharide 1,3-galactosyltransferase<br>lipopolysaccharide 1,2-glucosyltransferase waaJ<br>O-antigen ligase waaL<br>UDP-2,3-diacylglucosamine hydrolase lpxH<br>2-dehydro-3-deoxyphosphooctonate aldolase<br>3-deoxy-manno-octulosonate cytidylyltransferase<br>antimicrobial peptide resistance<br>phosphoheptose isomerase<br>lipopolysaccharide 1,3-galactosyltransferase<br>lipopolysaccharide 1,2-glucosyltransferase
**12**	O-Antigen nucleotide sugar biosynthesis	sty00541	14	UTP-glucose-1-phosphate uridylyltransferase<br>glucose-1-phosphate uridylyltransferase<br>UDP-glucose 4-epimerase gale<br>UDP-glucose 6-dehydrogenase UGDH<br>TDP-glucose pyrophosphorylase rfbA<br>glucose-1-phosphate thymidylyltransferase<br>dTDP-glucose 4,6-dehydratase<br>UDP-N-acetylglucosamine epimerase<br>dTDP-4-dehydrorhamnose reductase<br>lipopolysaccharide biosynthesis protein wecE<br>dTDP-4-dehydrorhamnose 3,5-epimerase<br>dehydratase RfbH<br>reductase RfbI<br>phosphomannomutase
**13**	Peptidoglycan biosynthesis	sty00550	2	Esterase<br>monofunctional biosynthetic peptidoglycan transglycosylase
**14**	Pantothenate and CoA biosynthesis	sty00770	7	3-methyl-2-oxobutanoate hydroxymethyltransferase<br>pantoate:beta-alanine ligase<br>pantothenate kinase<br>phosphopantetheine adenylyltransferase<br>holo-[acyl-carrier protein] synthase<br>4'-phosphopantetheinyl transferase<br>aspartate 1-decarboxylase

**15**	Polyketide sugar unit biosynthesis	sty00523	1	dTDP-4-dehydrorhamnose 3,5-epimerase
**16**	Monobactam biosynthesis	sty00261	2	aspartate-semialdehyde dehydrogenase<br>4-hydroxy-tetrahydrodipicolinate reductase
**17**	Streptomycin biosynthesis	sty00521	1	dTDP-4-dehydrorhamnose 3,5-epimerase
**18**	Benzoate degradation	sty00362	1	n-hydroxybenzoate hydroxylase
**19**	Aminobenzoate degradation	sty00627	1	flavin prenyltransferase
**20**	Nitrotoluene degradation	sty00633	2	oxygen-insensitive NAD(P)H nitroreductase<br>N-ethylmaleimide reductase
**21**	Methane metabolism	sty00680	30	Esterase frmB<br>formate dehydrogenase-O, major subunit<br>formate dehydrogenase-O subunit beta<br>phosphoenolpyruvate carboxylase<br>fructose 1,6-bisphosphate aldolase<br>6-phosphofructokinase isozyme<br>1-phosphofructokinase<br>trimethylamine-N-oxide reductase<br>chaperone protein TorD<br>acetate kinase<br>serine hydroxymethyltransferase<br>phosphate acetyltransferase<br>serine hydroxymethyltransferase<br>enolase<br>malate dehydrogenase<br>6-phosphofructokinase<br>acetyl-coenzyme A synthetase<br>phosphoenolpyruvate synthase<br>2,3-bisphosphoglycerate-dependent phosphoglycerate mutase<br>phosphoglycerate mutase 2<br>D-3-phosphoglycerate dehydrogenase<br>3-phosphoserine/phosphohydroxythreonine aminotransferase<br>phosphoserine phosphatase<br>glycerol metabolic protein<br>phosphoenolpyruvate synthase<br>2,3-bisphosphoglycerate-independent phosphoglycerate mutase
**22**	Cyanoamino acid metabolism	sty00460	1	periplasmic beta-glucosidase
